# Deep-learning-assisted Fourier transform imaging spectroscopy for hyperspectral fluorescence imaging

**DOI:** 10.1038/s41598-022-06360-y

**Published:** 2022-02-15

**Authors:** Cory Juntunen, Isabel M. Woller, Andrew R. Abramczyk, Yongjin Sung

**Affiliations:** 1 College of Engineering and Applied Science, University of Wisconsin-Milwaukee, Milwaukee, USA; 2 College of Health Sciences, University of Wisconsin-Milwaukee, Milwaukee, USA

**Keywords:** Wide-field fluorescence microscopy, Fluorescence spectroscopy, Fluorescence imaging, Imaging studies

## Abstract

Hyperspectral fluorescence imaging is widely used when multiple fluorescent probes with close emission peaks are required. In particular, Fourier transform imaging spectroscopy (FTIS) provides unrivaled spectral resolution; however, the imaging throughput is very low due to the amount of interferogram sampling required. In this work, we apply deep learning to FTIS and show that the interferogram sampling can be drastically reduced by an order of magnitude without noticeable degradation in the image quality. For the demonstration, we use bovine pulmonary artery endothelial cells stained with three fluorescent dyes and 10 types of fluorescent beads with close emission peaks. Further, we show that the deep learning approach is more robust to the translation stage error and environmental vibrations. Thereby, the He-Ne correction, which is typically required for FTIS, can be bypassed, thus reducing the cost, size, and complexity of the FTIS system. Finally, we construct neural network models using Hyperband, an automatic hyperparameter selection algorithm, and compare the performance with our manually-optimized model.

## Introduction

Fluorescence imaging allows for direct observation of various organelles in a biological specimen with high resolution and contrast. It typically uses fluorescent dyes which bind to different key targets/organelles of cells or fluorescent proteins (FPs) that are fused to protein targets in living cells^1^. A dichroic filter tuned for the characteristic excitation and emission bands of the fluorophore is typically required. For the observation of more than one fluorophore, a dichroic filter with multiple passbands or a set of filters mounted on a filter wheel is typically adopted. Many FPs commonly used in live-cell imaging have overlapping emission spectra, which limit the number of FPs that can be used simultaneously^2^. Hyperspectral imaging, which combines imaging and spectroscopy, allows for using a multitude of fluorophores with close emission peaks^3,4^. It also allows for accurate detection and quantification of target fluorescence signals in a tissue with highly autofluorescent background^5^.

For hyperspectral imaging with the spectral resolution of 10 nm or below, an acousto-optic tunable filter (AOTF), a liquid crystal tunable filter (LCTF), or Fourier transform spectroscopy (FTS) is typically combined with an imaging device. AOTF enables us to scan the entire visible wavelength range in several seconds or randomly access to any wavelength in the range^6^. LCTF is slower but can provide superior image quality^7^. AOTF provides a narrower spectral bandwidth (1.5–4.1 nm) than LCTF (4.5–19 nm)^7^. FTS is a preferred method when a high spectral resolution is required, the signal is weak, or both. In contrast to LCTF or AOTF, which passes only a narrow spectral band of interest, FTS uses the entire spectrum of interrogated light for each sampled data; thus, the sensitivity of FTS is very high^8,9^. This Fellgett’s advantage is especially useful when the target fluorescence signal is weak. For FTS, a Michelson interferometer or a Sagnac interferometer is typically used, which splits the interrogated light into two, and a series of intensity data is acquired for varying optical path differences (OPDs)^10^. The Fourier transform of the interferogram can be related to the spectral profile of the interrogated light. Fourier transform imaging spectroscopy (FTIS) combines FTS with an imaging device to measure the spectrum of each pixel of an image. FTIS has been used for various applications that require simultaneous use of multiple fluorophores. For example, it has been used to classify seven fluorophores with overlapping emission spectra in immunofluorescence-stained tissue samples^3^. FTIS is a gold standard method for spectral karyotyping, which uses a combination of 4-5 fluorophores to label 24 human chromosome pairs^11^.

One key disadvantage of FTIS is low throughput due to the large number of interferogram images that need to be collected to reconstruct the spectrum. Typically, more than 1000 images are acquired, which can take tens of seconds even with a high-speed camera. Several efforts have been made to reduce the sampling number with methods such as compressed sensing^12^. Since the interferogram measurements of FTIS are taken in the Fourier space, the signal measurement procedure for FTIS satisfies the incoherence property which is a requirement of compressed sensing^13,14^. Here, we show that deep learning can significantly reduce the required FTIS sampling number. As with FTS, FTIS records interferograms at uniform intervals of OPD; i.e., the distances between the moving mirror’s positions where the interferograms are recorded are assumed to be the same. However, due to environmental vibrations and translation stage error, the actual mirror position where each interferogram is recorded is different from the target position. To correct for this error, a reference laser (typically He-Ne laser) with a sharp peak at the known spectral position is inserted in the same beam path as the interrogated light, and its interferogram is used to find the true OPD (i.e., the actual mirror position) for each sampled data. The so-called He-Ne correction is crucial for accurate reconstruction of the spectrum using FTS, but requires additional optical components. Here we show that deep-learning-assisted FTIS obviates the He-Ne correction; thereby, it can reduce the complexity and footprint of the FTIS system. Deep learning-based approaches have been shown to outperform traditional methods in a wide array of fields including imaging^15^ and natural language processing^16^. For example, deep learning has been applied to improve the resolution of optical microscopy^17^, scanning electron microscopy^18^, and multispectral imaging^19^. The enhanced imaging performance has been used to accurately identify components of images that are indicative of specific diagnosis^15,20,21^.

## Materials and methods

### Materials

To demonstrate the proposed technique, we imaged bovine pulmonary artery endothelial cells labeled with three fluorescent dyes (MitoTracker Red CMXRos, Alexa Fluor 488 Phalloidin, and DAPI). The sample slide was purchased from Thermo Fisher (F36924). Then, we imaged 10 types (blue-green, green, yellow-green, orange, yellow, red, carmine, red-orange, crimson, and scarlet) of fluorescent beads with close emission peaks (Invitrogen FluoSpheres F21015 and F8891). For the training data, slides with individual bead types were prepared by centrifuging each sample type, adding FluorSave mounting medium (MilliporeSigma, 345789), mixing, and repeating one additional time. The bead samples were then pipetted onto a slide and sandwiched with a cover glass. For the test data, a combination of all bead types was prepared using the same method. Each sample was covered from light and given several hours for the mounting medium to solidify.

### FTIS optical system

**Figure 1:**
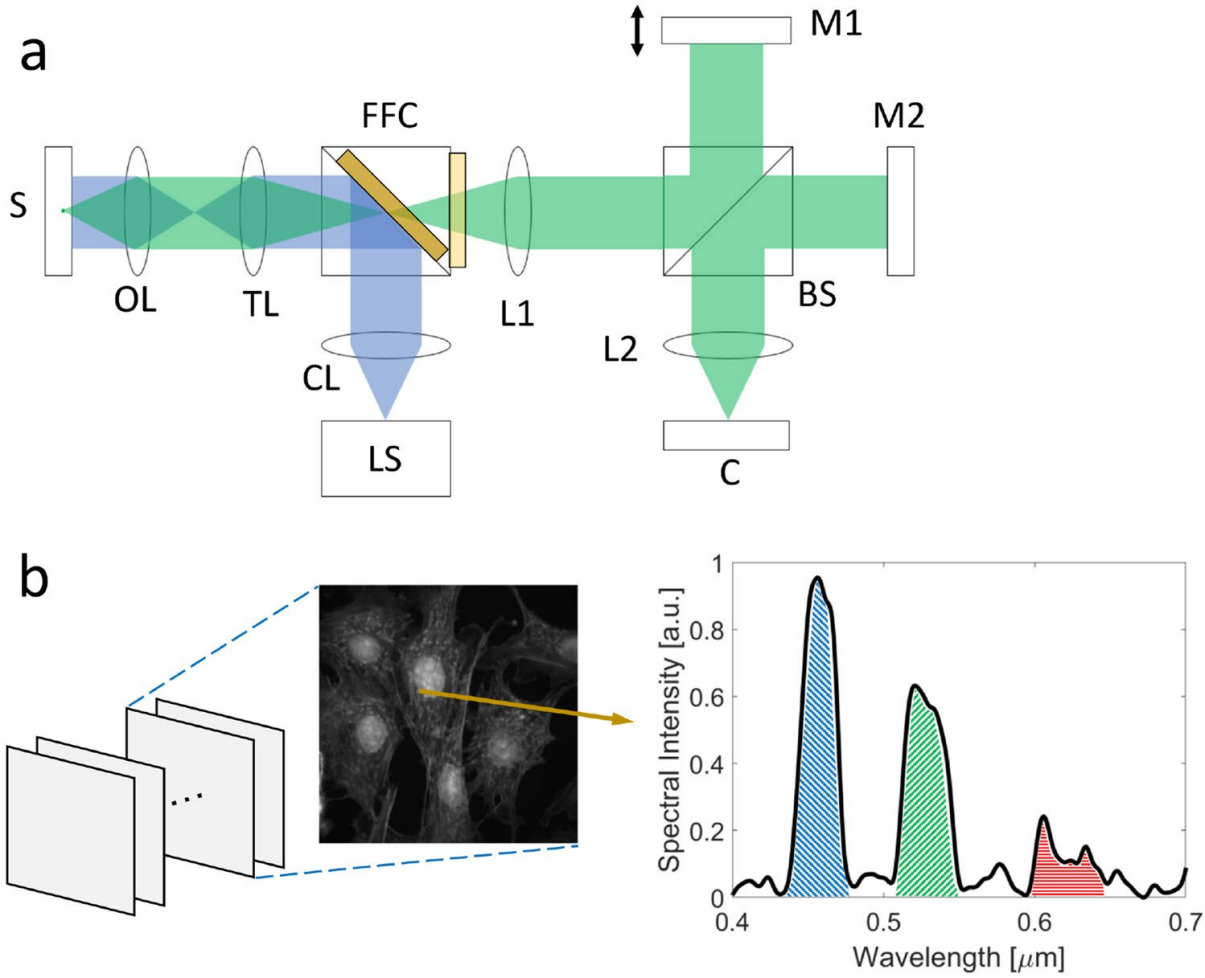
(**a**) Schematic diagram of the optical system showing the excitation beam path (blue) and emission beam path (green). LS: Light Source, CL: Collimating Lens, FFC: fluorescence filter cube, S: Sample, OL: Objective Lens, TL: Tube Lens, L1 and L2: Lenses, M1: Moving Mirror, M2: Stationary Mirror, BS: beam splitter, and C: Camera. (**b**) Interferogram image set and resulting spectral intensity. (**a**) and (**b**) created with Microsoft PowerPoint ver. 2111 Build 14701.20262 (https://products.office.com/en-us/powerpoint), graph in (**b**) exported from MATLAB ver. 9.10.0.1602886 (R2021a) (https://www.mathworks.com/products/matlab.html).

For the data collection, we have built a wide-field epi-fluorescence microscope equipped with a laboratory-built FTS module. Figure 1a shows a schematic diagram of the system. To image BPAE cells, we used three individually-controlled light-emitting diodes (Thorlabs, M385L2, M505L4, and M565L3) combined using two dichroic beam splitters (Thorlabs, DMLP425R and DMLP550R). The excitation light was delivered to the sample through a triple-band filter set (Semrock, DA/FI/TX-3X). The fluorescence light emitted from the sample was collected by the objective lens (OL) (Olympus, UPLFLN 100X). The numerical aperture was reduced to 0.6 to collect the fluorescence signal from the entire depth of the sandwiched BPAE cells. The tube lens (TL) of 100 mm focal length created an image at the intermediate image plane, which coincided with the input plane for the FTS module. The FTS module was built upon a Michelson interferometer with one of the mirrors mounted on a motorized translation stage (Physik Instrumente, M-227.25). Two lenses (L1 and L2) of the same focal length (150 mm) in a 4-f telecentric configuration relayed the image from the intermediate image plane to the image plane where the camera was located. To record the images, we used an electron-multiplying charge-coupled device (EMCCD) camera (C) (Andor, iXon Ultra 888). The FTS module recorded a multitude of images for varying OPDs, i.e., for different locations of the moving mirror. Because the wavelength of a He-Ne laser fell in one of the fluorescence emission bands, we used a diode laser of the wavelength 488 nm (Coherent, OBIS 488 nm LS 60 mW) to record the actual mirror position. The intensity of the reference interferogram was monitored using a silicon photodiode (Thorlabs, SM1PD1A). To minimize contamination of the fluorescence images by scattered light photons of 488 nm, we operated the diode laser at a minimum power level (1 mW) and installed a notch filter (Thorlabs, NF488-15) in front of the EMCCD camera. The rejected band did not overlap with the emission bands of the fluorescence filter set. The control of the translation stage as well as the acquisition of the fluorescence images and the reference interferogram were controlled using a Labview program (National Instruments, version 15). To image 10 types of fluorescent beads, we used a high-power, white LED (Thorlabs, SOLIS-3C) as the light source together with a long-pass dichroic filter (Thorlabs, DMLP425R) and an emission filter (Thorlabs, FELH0450). To record the bead images, we used a scientific Complementary metal-oxide-semiconductor (sCMOS) camera (pco.edge 5.5).

### Data acquisition

Before the experiment, the zero OPD position, where the interferogram had the maximum value, was found. To image BPAE cells, the power of each excitation LED was adjusted to produce about the same fluorescence intensity levels for all the fluorophores. For each field of view (FOV), 1000 interferogram images were recorded while moving the translation stage in steps of 50 nm. This step size corresponds with 100 nm in terms of the OPD. The images were recorded with the camera EM gain of 300 and the exposure time of 0.01s. Simultaneously capturing the images with the camera, the reference interferogram was collected with a photodiode. Each measurement took 23 seconds per set of 1000 images. A total of 30 sets of images (i.e., FOVs) were collected. For the fluorescent beads, the power of the excitation LED and the exposure time of the camera were adjusted to prevent pixel saturation for the scarlet bead, which produced the strongest fluorescence intensity. The same setting was used for all the other bead types. In each FOV there were a minimum of three beads. For the training data, interferograms from 10 FOVs were collected for each fluorescent bead type. For the test data, interferograms from twenty FOVs with mixed fluorescent beads were collected.

### Data preprocessing

**Figure 2:**
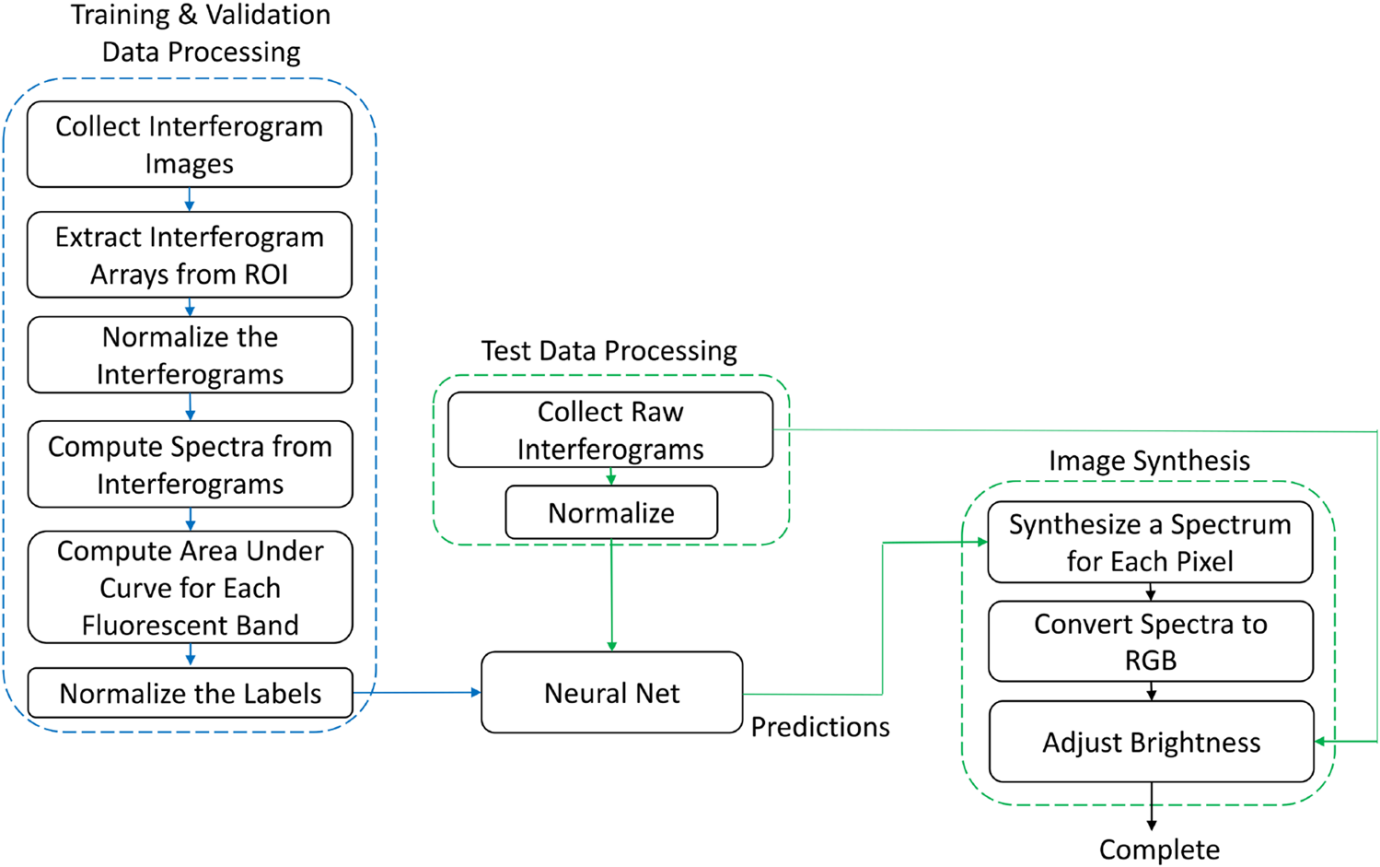
Data processing flowchart starting with collected interferogram images. Training data is processed by computing the spectra, and finding the area under the curve of each fluorescence emission band. The neural network is trained, and then used to predict and synthesize an image from an unknown sample. Training process is shown in blue, while the testing process is shown in green. Validation process followed training process except with a sample which was not trained on. Created with Microsoft PowerPoint ver. 2111 Build 14701.20262 (https://products.office.com/en-us/powerpoint).

A flow chart of the data processing procedure is shown in Fig. 2. The training data flow is shown on the left side of the figure. For each FOV, we selected the sample region using a binary mask, which was obtained by applying a threshold to the maximum projection of the raw interferogram images. Then, for each pixel in the sample region, we extracted the raw interferogram, and the maximum intensity was saved for later use. Each interferogram was detrended, and normalized so that the peak was the center, with a mean of 0.5 and a maximum of 1.

For the BPAE cell imaging, we trained a neural network (NN) to predict the normalized channel intensity (NCI), the area under each fluorescence emission band divided with the total area for all the three emission bands, which is shown in Fig. 1b. The ground truth NCI values were computed from the interferograms using the conventional FTS method (including He-Ne correction). For each channel (i.e., emission band), 20,000 interferograms producing the highest NCI values were selected, which resulted in 60,000 interferograms from each FOV. Out of 30 FOVs, the datasets for 28 FOVs were used for training. The dataset for two remaining FOVs were set aside for validation and testing. The training data was randomly mixed so that each mini batch contained data from multiple FOVs, multiple locations on each sample, and different fluorescent signal types. The training data was augmented by adding three types of error: the peak location was shifted from the center by an amount randomly sampled between − 3 and 3, the mean was shifted from 0.5 by an amount sampled from a normal distribution with a mean of zero and a standard deviation of 0.05, and noise sampled from a normal distribution with a mean of zero and a standard deviation of 0.05 was added to each point in each interferogram.

For the classification of 10 types of fluorescent beads, 1000 randomly selected pixels from each training sample FOV were saved, resulting in 10,000 interferograms for each fluorescent bead, 100,000 total. The average spectrum of each fluorescent bead was computed using the He-Ne corrected data and the NUFFT for the range of 400–800 nm. Because the training dataset for each bead type was acquired separately, the ground truth label is known. To determine the ground truth labels of the mixed test data, the MSE between the test data pixel spectrum and each of the 10 averaged training data spectrum was calculated, and the dye type producing the lowest MSE was assigned to the pixel. This method produced over 99.99% accuracy when it was applied to the training dataset. Once all of the labels were computed, the He-Ne-corrected interferograms were discarded, and only the raw interferograms were used by the NN.

### Deep learning model

**Figure 3:**
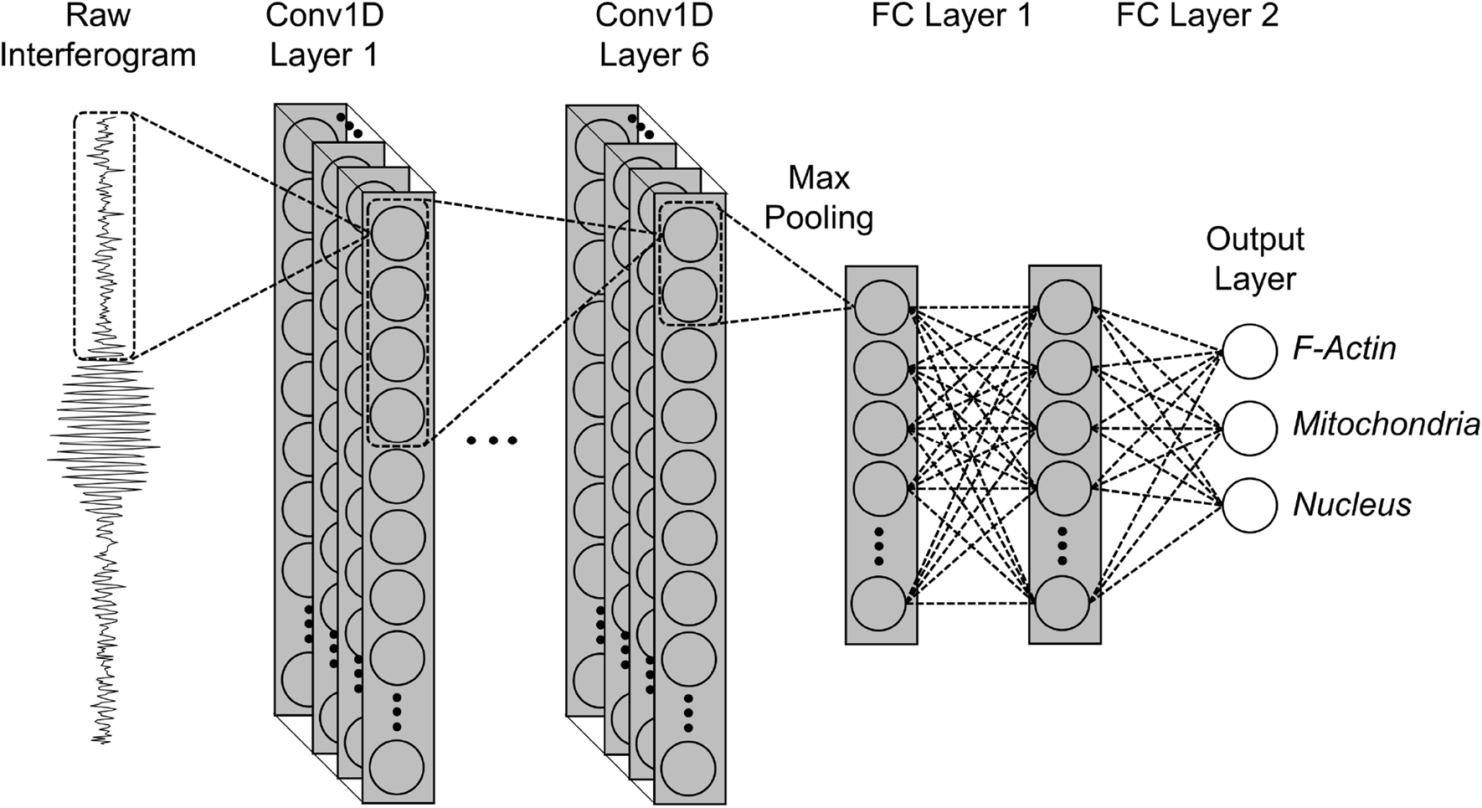
Schematic of 1DCNN used for this study, which is comprised of six 1D convolutional layers followed by a max pooling layer, and two fully connected layers. The raw input data consists of an interferogram with 1000-50 sampled points each. The values at the output predict the amount of each type of fluorescence signal in the pixel. Created with Microsoft PowerPoint ver. 2111 Build 14701.20262 (https://products.office.com/en-us/powerpoint).

Figure 3 shows a schematic of the 1D convolutional neural network (1D CNN), which was used for the BPAE cell imaging. After each convolutional block, there is a max pooling layer which outputs the maximum of each neighborhood. After the max pooling layer of the final convolutional block, the model is flattened and connected to one or more fully connected “dense” layers providing enough capacity for the model to correctly represent the function before reaching the output layer. The output layer consists of 3 fully connected nodes with sigmoid activation to predict the normalized channel intensity for each of 3 fluorescence emission bands. For the classification of 10 types of fluorescent beads, the number of nodes in the output layer was increased to 10. Although we tested several configurations, using several layers of small kernels allowed us to detect more complex features at a lower cost than using larger kernels, which is consistent with the previous works, for example, Simonyan and Zisserman^22^. The commonly used ReLU activation function was applied at each of the hidden layers. ReLU activation is widely used for deep CNNs as it introduces nonlinearity while being more computationally efficient than other nonlinear activation functions such as tanh^23,24^. Regularization reduces validation and test loss while sacrificing training loss leading to better generalization. Here, we selected to use dropout and L2 regularization, which is often referred to as “weight decay”, with a constant value in each layer. L2 regularization penalizes large weights without leading to additional model sparsity, which results from L1 regularization^25^. The Adam adaptive learning rate optimization algorithm was selected for weight optimization, which is known to perform well with stochastic gradient descent^26^. The loss for the cost function was selected to be based on mean absolute error (MAE) instead of mean squared error (MSE), because MAE-based loss punishes smaller errors in prediction harder than MSE-based loss, which disproportionately punishes larger prediction errors. With our BPAE cell dataset, the models trained with MAE-based loss tended to synthesize images closer to the ground truth.

### Training of NN with 1D interferogram

The 1D CNN was trained using the standard training procedure shown in the lower middle area of Fig. 2. For the BPAE cell imaging, training data is fed to the NN, which outputs predictions. These predictions are compared with the ground-truth NCI values to compute the loss, and the weights of the 1D CNN are optimized to minimize the loss. Our training set contained 1,680,000 interferograms from 28 FOVs. Each epoch, or time the 1DCNN trains on the mini batches covering the entire data set, the MAE and loss for training data were calculated and written to a separate file; the same was done for the validation set which consisted of 30,000 interferograms (10,000 producing the highest NCI values for each channel) from a separate FOV. These values are evaluated by the early stopping algorithm. Early stopping is important as it functions as a source of regularization in a synergistic way alongside L2 regularization^17^. The early stopping algorithm observes the loss of the validation data and the epoch number. The training stops if either of the following conditions has been met: (1) the current epoch has reached the maximum number of desired epochs (2) the loss at the current epoch has not decreased below the minimum recorded loss in a set number of epochs, which is referred to as the patience. We used the maximum iteration number of 100 and patience of 5. When an early stopping condition has been met, the training stopped, and the model resulting in the lowest validation MAE was saved. For the training and testing of NN, we used a workstation with two GPUs (NVIDIA Quadro P6000). The MirroredStrategy function built in to TensorFlow was used for synchronous training across the multiple GPUs on a single workstation.

For the classification of the 10 fluorescent beads, the same procedure was used to train the neural network except for a few things. First, the main difference in the architectures was that the output layer had 10 output nodes with the Softmax activation function. Second, the classification accuracy was monitored instead of the MAE. Finally, it was observed that the validation accuracy was much higher when the cost function used MSE as opposed to the MAE used in the BPAE cell label regression when testing a few architectures. For this reason, MSE was used for the cost function in the NN classifying the 10 fluorescent beads.

### Hyperparameter selection

**Figure 4:**
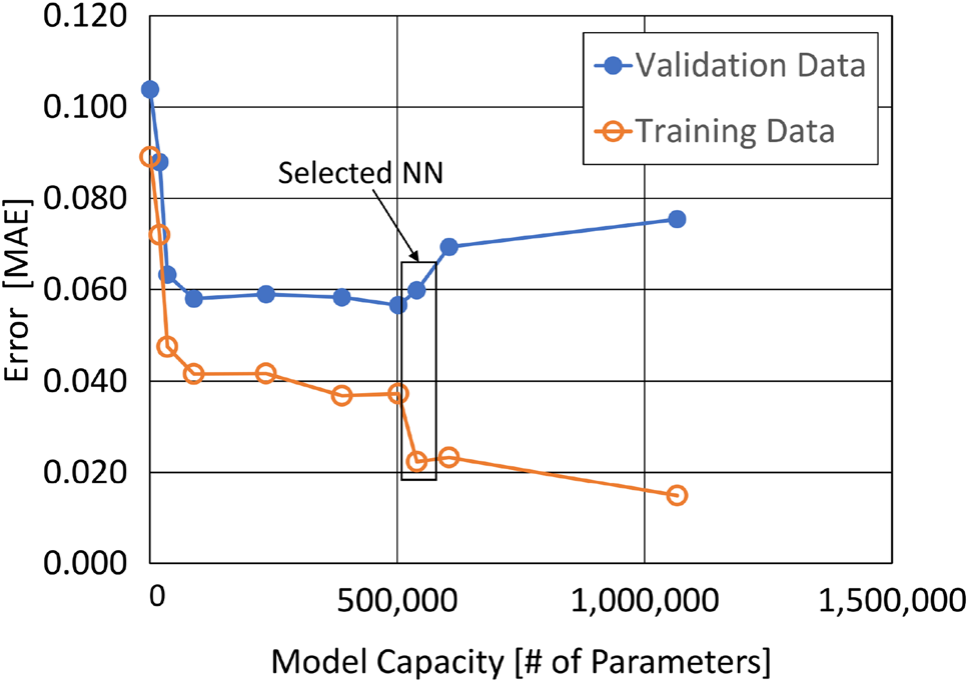
Error vs model capacity of 1DCNNs with manually selected hyperparameters, without regularization.

The hyperparameters for our NN models were manually selected while monitoring the MAE or MSE values for the training data and validation data as shown in Fig. 4. For each interferogram sampling case, the model capacity (the number of trainable weights and biases) was increased until overfitting was observed. More specifically, each initial model started with only one convolutional layer consisting of a small number of filters and a small kernel size, a max pooling layer with large pool size, and the output layer. Each new model introduced a new layer, more parameters per layer, or a decrease in pool size in a nonuniform fashion, intending to make small changes and slightly increase the number of parameters. Filters per convolutional layer ranged from 32 to 128, kernel size ranged from 4 to 8, pool size ranged from 100 to 4, and dense layer nodes ranged from 256 to 3. Once the model with suitable capacity was selected, L2 regularization was increased until the difference between training and validation loss was minimized. For comparison, we have also generated NNs using Hyperband, an automatic hyperparameter selection algorithm which uses random search and successive halving^27^. The process starts with a multitude of NN architectures trained in search space for a small number of epochs. The best performing networks are trained further while the poor performing networks are abandoned. This process continues until the best network and corresponding set of hyperparameters are chosen.

### Image synthesis

For the FTS-computed spectrum, the cell image was synthesized by converting each wavelength to an RGB value and then taking the sum of the RGB values weighted by the spectral intensity. The outputs from the NN, which are labeled as “Predictions” on the flow chart in Fig. 2, represent signal weight between 0 and 1 of each fluorescent band region. These predictions were each multiplied with their respective average spectrum (obtained from the training data) and combined, resulting in a synthesized spectrum for each pixel. The maximum intensity of raw interferogram saved at each pixel was used to adjust the scale of the synthesized spectrum. The adjusted spectrum for each pixel was converted to RGB values the same way as described earlier, resulting in an RGB image.

## Results and discussion

Providing the fluorescence spectrum for each pixel, hyperspectral imaging allows us to use various fluorophores with close emission spectra and distinguish target fluorescence signals from autofluorescence background. With the superior spectral resolution of FTIS, we can increase the number of fluorophores that can be simultaneously used, thereby increasing the imaging throughput and obviating the need for sample washing. Here we show our deep learning-based approach can increase the throughput by an order of magnitude while minimally sacrificing the accuracy of measurement.

**Figure 5:**
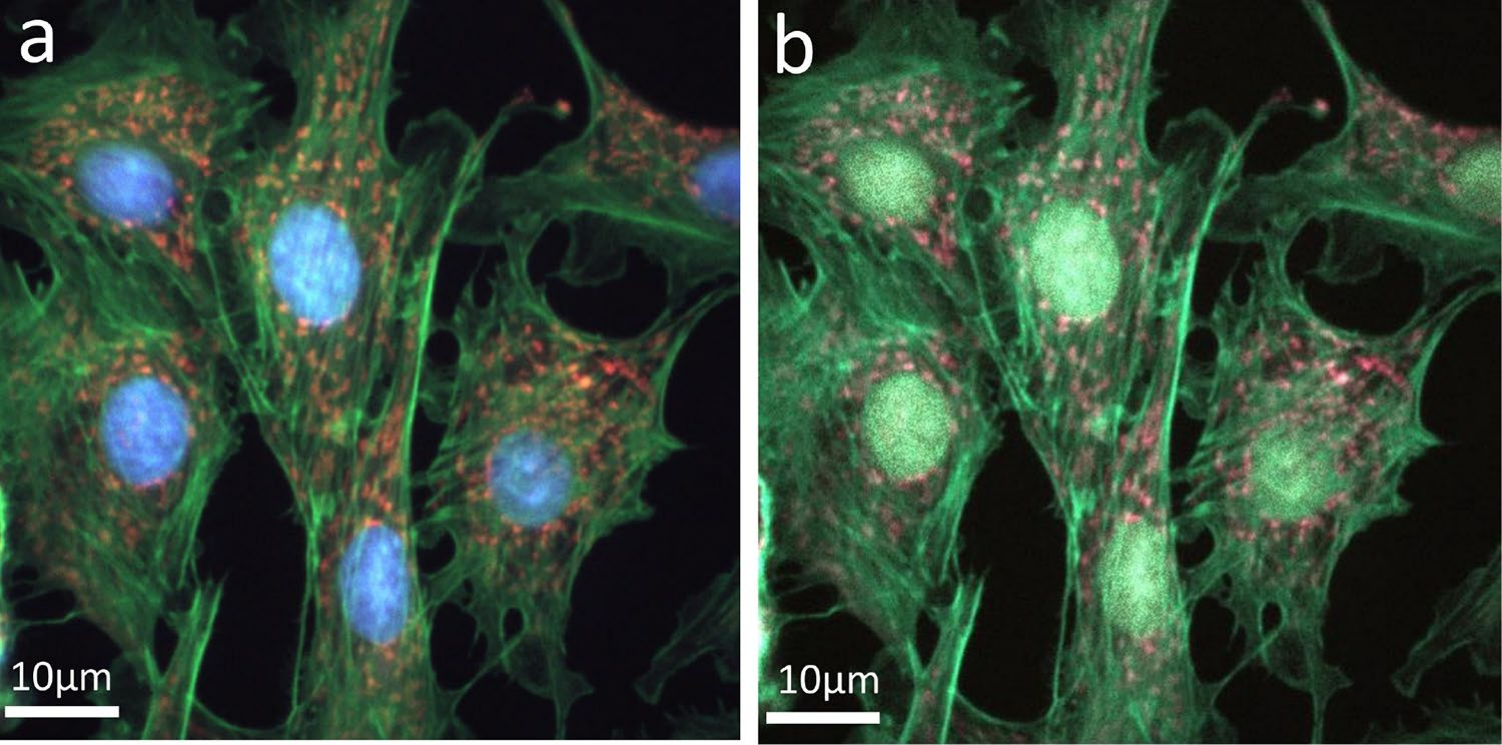
Synthesized fluorescence images using conventional FTS with He-Ne correction for various sampling numbers (N): (**a**) N = 1000, and (**b**) N = 50. Scale bars: 10 μm.

**Figure 6:**
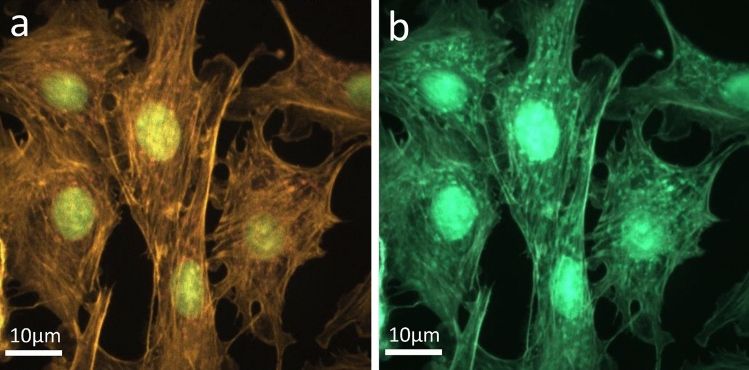
Synthesized fluorescence images using conventional FTS without He-Ne correction for various sampling numbers (N): (**a**) N = 1000, and (**b**) N = 50. Scale bars: 10 μm.

Figure 5 shows the images synthesized with FTS for different sampling numbers: (a) 1000, and (b) 50. The raw interferogram for each pixel was He-Ne corrected before being processed with the FTS algorithm. Figure 5a is the ground truth that we use for comparison. Figure 5b, reconstructed from 50 sampled points, shows the image completely lost the ability to distinguish between the blue DAPI (nucleus) and green Alexa Fluor 488 Phalloidin (F-actin) fluorescent dyes. Figure 6 shows the FTS-synthesized images without the He-Ne correction. For N = 1000 (Fig. 6a), the nucleus is shown in an incorrect color, and the F-actin and mitochondria are indistinguishable. For N=50 (Fig. 6b), all three fluorescent dyes are indistinguishable. Comparing the images with those in Fig. 5, the importance of He-Ne correction in the conventional FTS can be clearly seen.

**Figure 7:**
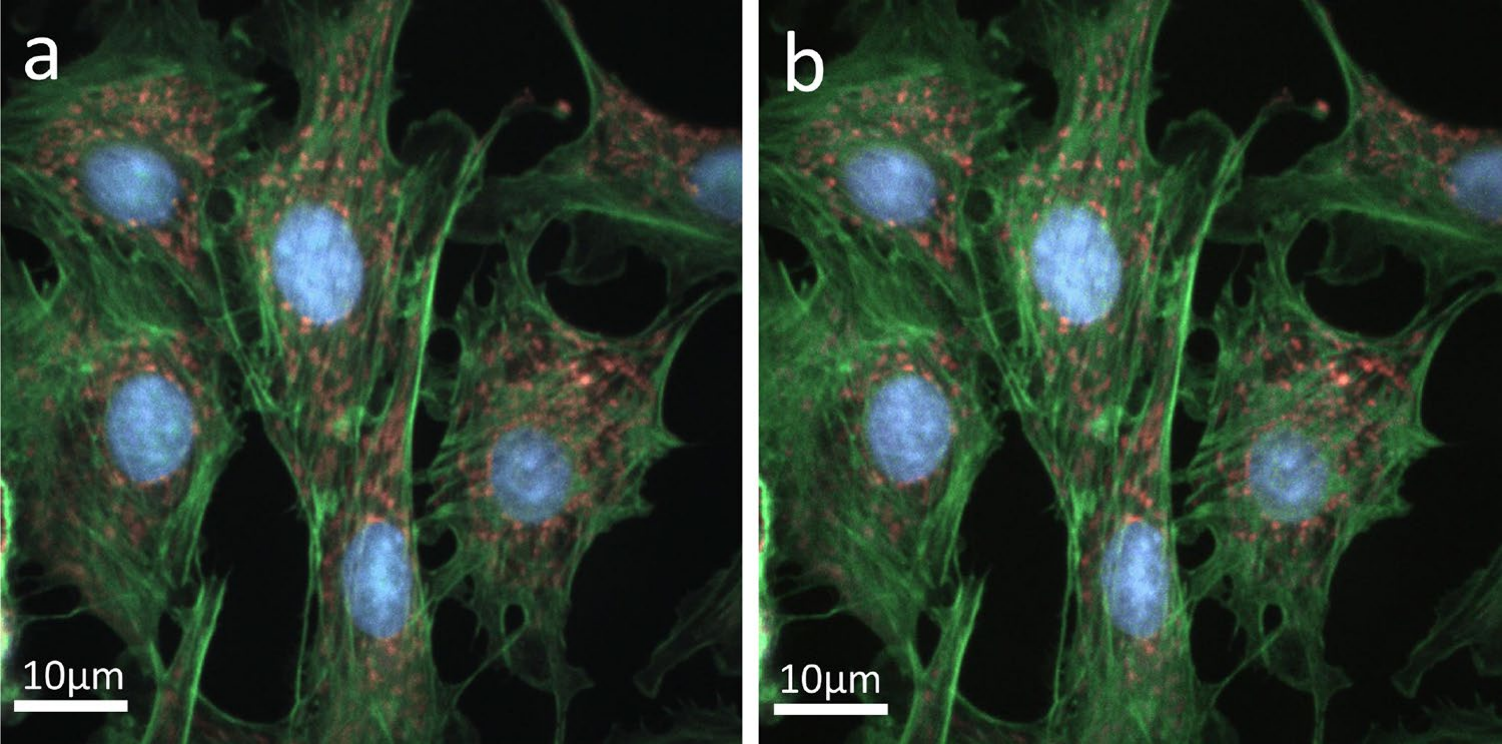
Synthesized fluorescence images using deep learning without He-Ne correction for various sampling numbers (N): (**a**) N = 1000, and (**b**) N = 50. Scale bars: 10 μm.

Figure 7 shows the images synthesized with the NN described earlier. The interferograms used for the training, validation, and testing of NN have not been corrected with the He-Ne data. Even though the He-Ne correction was not applied, the NN-synthesized image shown in Figure 7(a), which corresponds to N=1000, looks very similar to the ground truth, Fig. 5a. The image shown in Fig. 7b is also almost the same as the ground truth except for some speckles. This is remarkable considering that it was synthesized from 20 times less data than the ground truth and without the He-Ne correction.

**Figure 8:**
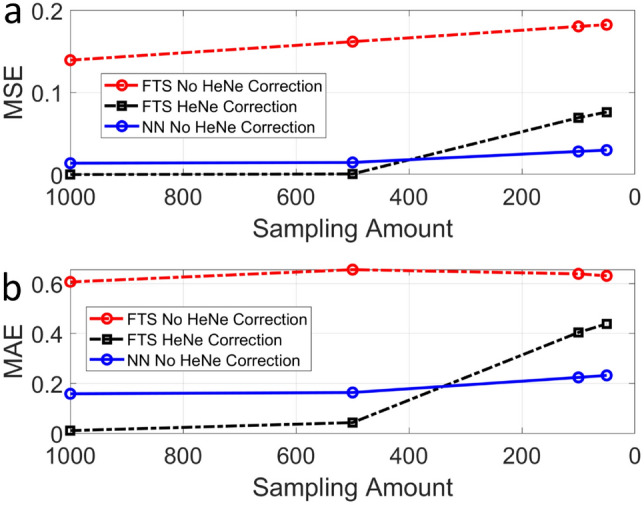
Comparison of traditional FTS with He-Ne correction, FTS without He-Ne correction, and NN without He-Ne correction for varying sampling amounts. MSE: mean squared error; and MAE: mean absolute error.

Figure 8a shows the MSE with varying sampling amounts for the various synthesis methods: FTS without He-Ne correction, FTS with He-Ne correction, and NN without He-Ne correction. The FTS without He-Ne correction method starts heavily degraded with a high MSE (> 0.14) even for N = 1000. The MSE of FTS reconstruction with He-Ne correction is very low for N = 1000; however, it quickly degrades to near 0.08 as the sampling number decreases. In contrast, the NN without He-Ne correction has some small MSE at full interferogram sampling of N = 1000, and the MSE stays below 0.04 as the sampling number decreases. For N = 50, the MSE for the NN without He-Ne correction is less than half of the corresponding FTS with He-Ne correction. While the images computed by FTS with and without He-Ne correction are heavily degraded with less sampling, the images synthesized by the NN remain intact at the sampling amount of N = 50. The limit of our approach appears to be at N=50, which is reducing the sampling number by 95%.

**Figure 9:**
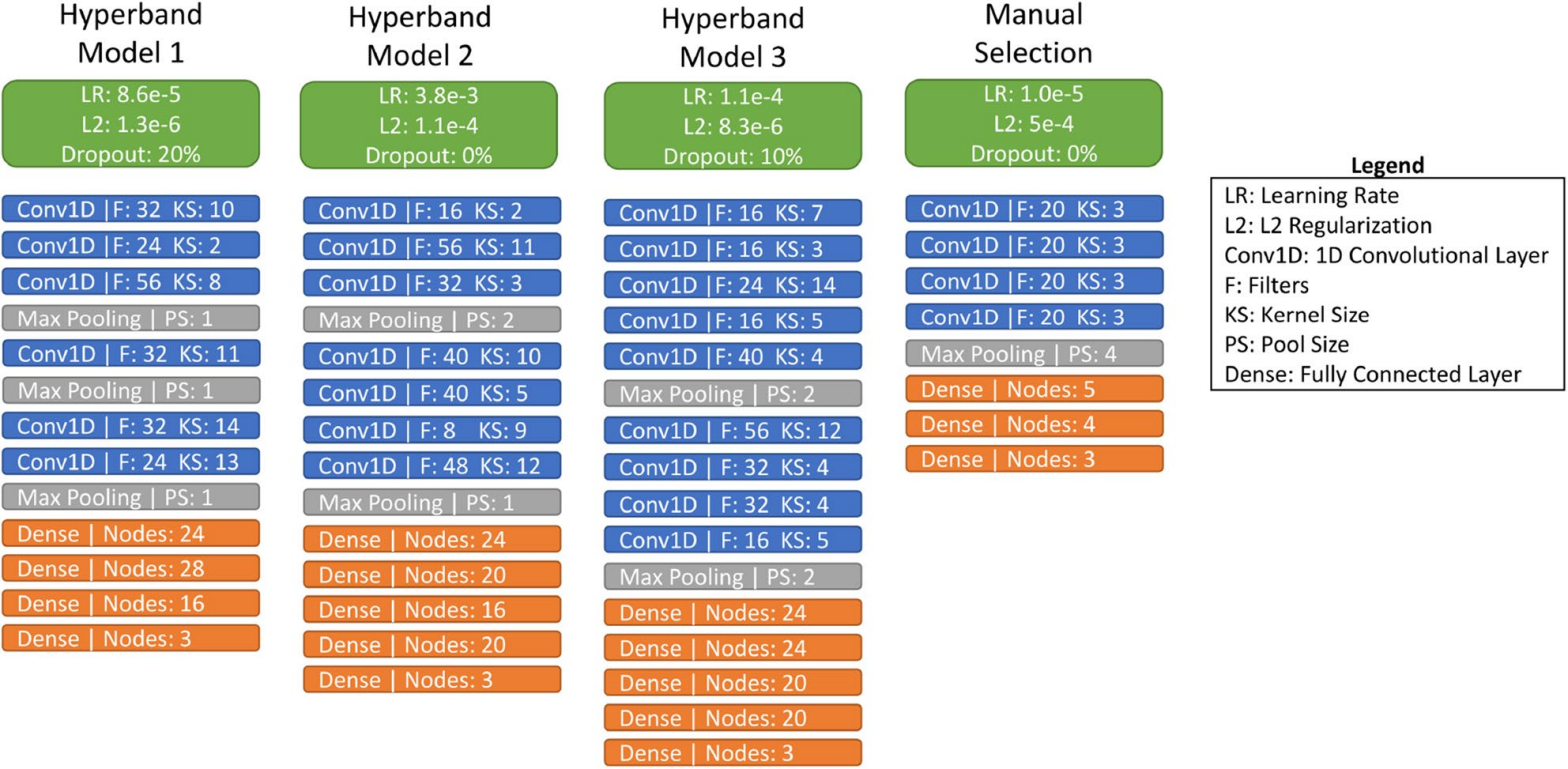
Architectures of the top 3 performing models selected by Hyperband and the architecture resulting from manual hyperparameter selection.

The results presented earlier were obtained using a manually optimized NN. For comparison, we built NNs using Hyperband, an automatic hyperparameter selection algorithm. Figure 9 shows the hyperparameters of the top 3 Hyperband models, which have a few key differences. Model 1 does not use pooling, which may reduce the ability to detect translations in patterns^25^, but uses the highest dropout rate of 20%. Model 2 is the only model to use L2 regularization without dropout. To compensate for omitting dropout, the L2 regularization in Model 2 is two orders of magnitude higher than the other models which also feature dropout. Model 3 features 2 standard convolutional blocks, each with max pooling which may lead to the ability to detect pattern translation^25^. The manually selected model uses only one convolutional block consisting of 4 convolutional layers and a max pooling layer with the largest pool size of the 4 models. It also uses significantly fewer nodes in the fully connected layers. Using the models shown in Fig. 9, we applied k-fold cross validation with 10 folds using only the training and validation sets.

Table 1 shows a table of k-fold cross validation results for the N=50 case with the models found using Hyperband compared to the manually selected model. From Table 1, we see that Model 3 has the lowest validation error (MAE) of all the models. For this N = 50 case, the Hyperband model search space was between 1,083 and 1,015,075 trainable parameters. The model resulting from manual hyperparameter selection consisted of only 4,460 trainable parameters, an order of magnitude below all three models selected by Hyperband. This manually selected model resulted the highest mean k-fold cross validation error out of the 4 models; however, its test error is greater than the best performing model, Model 3, only by 8%. In contrast, Models 1 and 2 produced about the same validation error as Model 3 but significantly higher test error: 15% and 36%, respectively. The good generalization of the manually selected model is also reflected in the smallest standard deviation for the validation error and may be attributed to the small number of parameters (i.e., low capacity). Since the Hyperband models have an order of magnitude more parameters than the manually selected model, and more than double the standard deviation of validation error, they appear to be memorizing some of the training sets. Therefore, we proceeded with the manually selected model.

**Table 1:** K-fold cross validation results (MAE) of the top 3 performing model architectures selected by the Hyperband hyperparameter optimization algorithm compared with the model with manually selected hyperparameters.

	Hyperband (1,083 – 1,015,075 parameters)			Manual selection
	Model 1	Model 2	Model 3	
Number of parameters	86,879	73,855	57,903	4,460
Validation error (MAE)	0.0405 ± 0.0017	0.0432 ± 0.0027	0.0393 ± 0.0014	0.0589 ± 0.0006
Test error (MAE)	0.0976	0.1157	0.0852	0.0920

**Figure 10:**
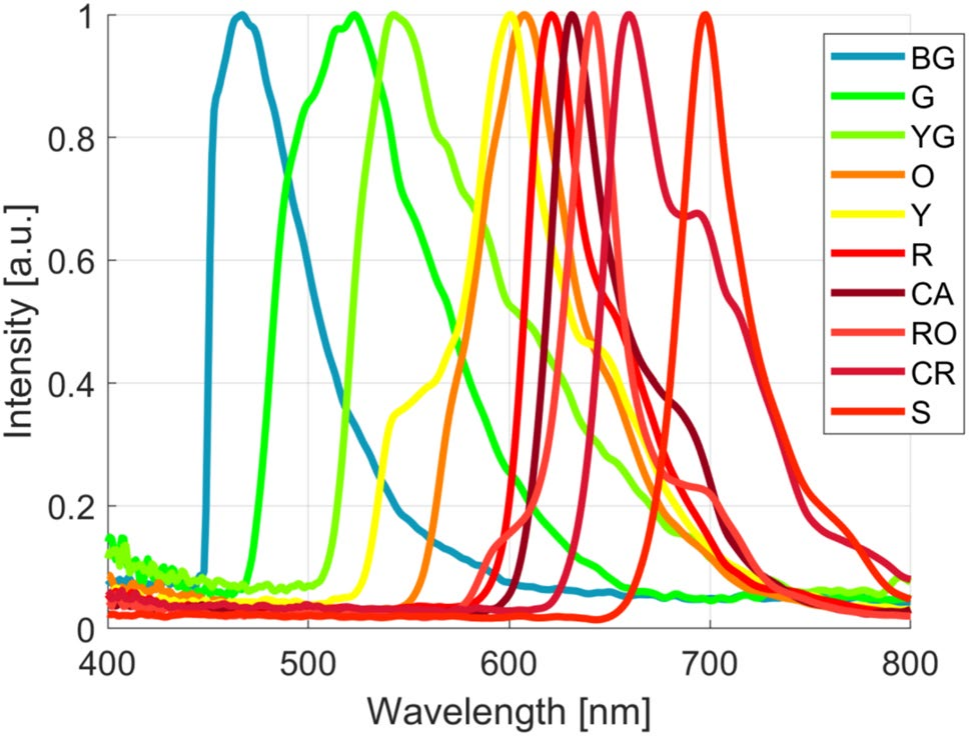
Average spectra of each fluorescent bead sample using full sampling of N=1000 and He-Ne correction. BG: blue-green, G: green, YG: yellow-green, O: orange, Y: yellow, R: red, CA: carmine, RO: red-orange, CR: crimson, and S: scarlet.

**Figure 11:**
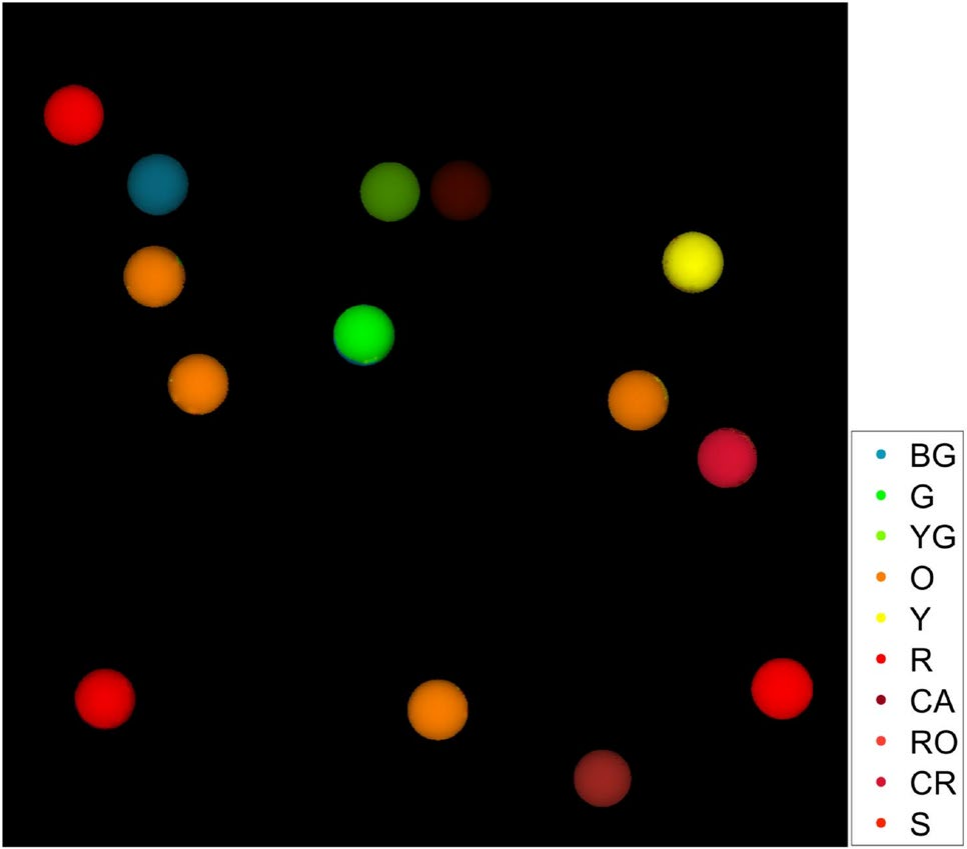
Synthesized image of 10 types of fluorescent beads using classification predictions by the convolutional neural network with N=100 non He-Ne corrected interferograms at each pixel.

To further demonstrate the performance of deep-learning-assisted FTIS, we demonstrated the technique using 10 fluorescent microspheres with close emission peaks and overlapping spectra shown in Fig. 10. For the N = 50 case, the best classification accuracy that was achieved was 85%. This low accuracy is attributed to the close emission spectra of some beads. We were able to achieve a very high classification accuracy of 97.8% for N = 100, which is 10 times less sampling than conventional FTS. The confusion matrix in Table 2 shows that the main source of error comes from the yellow bead pixels being mistaken for the orange bead pixels, and the red-orange bead pixels being confused with the carmine bead pixels. Looking at the average spectra of these beads shown in Fig. 10, the yellow and orange spectra peak locations are indeed very close relative to the other fluorescent dyes. For the carmine and red-orange spectra, they are close, but also have similar bandwidths which could have contributed to the problem. Regardless, the 1DCNN is able to very accurately classify 8 of the 10 fluorescent beads, and acceptably classify all 10 fluorescent beads with only 1/10th of the data sampling amount. Figure 11 shows a test sample containing each type of fluorescent bead and the pixel classification of each pixel interferogram for the N = 100 case. We observe that the beads are very accurately classified, and the only error appears to be near the edges of some beads.

The significant reduction of interferogram sampling by 10 to 20 times is comparable to the performance demonstrated with compressed sensing, which requires 1/16 of data traditionally needed^28^ or 1/9 of random samples from the original dataset^29^. Reducing the sampling size allows FTIS-based approaches to be readily used for hyperspectral fluorescence imaging, allowing more fluorescent dyes to be used including dyes with close emission spectra. Obviating the need for He-Ne correction, we can eliminate several optical elements, and thereby reduce the cost, size, and complexity of the FTIS system.

**Table 2:** Confusion matrix (%) of final 1DCNN for classification of 10 types of fluorescent beads from non He-Ne corrected interferograms with N=100 sampling.

N=100		Prediction									
		BG	G	YG	O	Y	R	CA	RO	CR	S
Actual	BG	100	0	0	0	0	0	0	0	0	0
	G	0	100	0	0	0	0	0	0	0	0
	YG	0	0	100	0	0	0	0	0	0	0
	O	0	0	0	100	0	0	0	0	0	0
	Y	0	0	0	12	88	0	0	0	0	0
	R	0	0	0	0	0	100	0	0	0	0
	CA	0	0	0	0	0	0	100	0	0	0
	RO	0	0	0	0	0	0	10	90	0	0
	CR	0	0	0	0	0	0	0	0	100	0
	S	0	0	0	0	0	0	0	0	0	100

We observed that the MAE loss worked better for the reconstruction of BPAE cells, which required a regression-type NN prediction. However, the MSE loss worked better for the classification of 10 bead types. The superior performance demonstrated with the MAE loss may be attributed to its lower sensitivity to outliers; however, we would need a more extensive study to confirm this, which is left as our future study. The NN prediction is applied to each pixel and capable of distinguishing multiple fluorophores mixed at different concentrations in the pixel volume. Changing the labeling protocol would not affect the NN accuracy, unless it significantly alters the emission spectrum of each fluorophore. The noise that can affect the accuracy of NN most is the Poisson noise due to low fluorescence signal. Noteworthy, FTIS provides higher signal-to-noise ratio than LCTF- or AOTF-based hyperspectral imaging due to the Fellgett’s advantage. The optical aberration may affect the spatial registration of the fluorescence signal; however, it will not affect the NN prediction. It is well established that the He-Ne correction can compensate for the stage error and environmental vibrations in the FTS reconstruction. We also confirmed this by comparing the MSE/MAE values of FTS with and without He-Ne correction (Fig. 8). For all the sampling number cases, the NN produced MSE/MAE values that are much lower than those for FTS without He-Ne correction. This confirms that the NN is more robust to the stage error and environmental vibrations than the FTS without He-Ne correction. A more systematic study on the relationship between the actual noise level and the accuracy of FTS as well as NN is left as our future study.

## Conclusion

In this paper, we have demonstrated hyperspectral fluorescence imaging by combining deep learning and FTIS. The image synthesized by the NN with a 10-20 times reduction in sampling accurately matched the ground truth image. Using triple-labeled bovine pulmonary artery endothelial cells and 10 types of fluorescent beads with close emission peaks, we demonstrated the capabilities of our approach. While greatly reducing the required sampling, we also bypass the need for He-Ne correction, eliminating several optical elements which reduces the cost, size, and complexity of the FTIS system. The developed system can be used in a wide range of applications where several fluorescent dyes with close emission spectra must be used, with much higher throughput.
